# Effect of Temperature on Thermal Oxidation Behavior of Ti-6Al-4V ELI Alloy

**DOI:** 10.3390/ma17164129

**Published:** 2024-08-20

**Authors:** Krzysztof Aniołek, Adrian Barylski, Jan Rak

**Affiliations:** Faculty of Science and Technology, Institute of Materials Engineering, University of Silesia, 75 Pułku Piechoty 1A, 41-500 Chorzów, Poland; adrian.barylski@us.edu.pl (A.B.); jan.rak@us.edu.pl (J.R.)

**Keywords:** Ti-6Al-4V ELI, thermal oxidation, micromechanical properties, wear, friction, surface roughness

## Abstract

In this paper, the morphological, micromechanical and tribological characteristics of the Ti-6Al-4V ELI alloy after thermal oxidation (TO) were identified. TO was carried out at temperatures of 848 K, 898 K and 948 K over a period of 50 h. Microscopic examination revealed that an increase in temperature resulted in an improved uniformity of coverage and an increased oxide grain size. Micromechanical tests showed that TO of the Ti-6Al-4V ELI alloy led to an increase in hardness and deformation resistance. Following oxidation, a decrease (by approximately 10–22%) was observed in the total mechanical work of indentation, W_total_, compared to the as-received material. The formation of protective oxide films on the Ti-6Al-4V ELI alloy also led to the improvement of tribological characteristics, both when tested under dry friction conditions and in Ringer’s solution. The sliding wear resistance increased with an increase in the oxidation temperature. However, a greater degree of wear reduction (by approximately 30–50%) was found for the lubricated contact in comparison with the dry friction tests. Surface roughness also increased with the increase in temperature.

## 1. Introduction

Titanium and its alloys are of interest to the chemical, aerospace, marine and medical industries due to their favorable properties, which include both high specific strength and crack and corrosion resistance [1,2,3]. Based on their crystallographic structure, titanium alloys are divided into α, β and α + β, each type offering a different combination of strength, anticorrosion properties and machinability. The wide variety of titanium alloys and their varied properties allow the fulfilment of many specific application requirements [4]. Despite its many benefits, titanium materials are also characterized by certain limitations. The cost of manufacturing and processing titanium and its alloys is higher compared to other engineering materials, which is predominantly due to the complex manufacturing process [5,6]. Besides production limitations, titanium-based materials can also generate a variety of operational problems. In tribological applications, particularly under sliding or rolling friction conditions, these materials are subject to rapid wear and tear, which significantly shortens their service life or considerably limits their applicability in friction couples [7,8]. During tribological operation, these materials are characterized by a high and unstable friction coefficient, which leads to negative effects and, eventually, may result in seizure. The increased risk of seizure is a result of the propensity of titanium and its alloys to adhere (cling) to other metals [9].

The affinity to atmospheric gases (oxygen, nitrogen and hydrogen) is an important characteristic of titanium and its alloys. This characteristic imposes the requirement for a protective atmosphere or even a vacuum in the various technological processes to which titanium materials are subjected and limits their use at elevated temperatures. However, this seemingly adverse characteristic offers a great potential for shaping oxide films on titanium-based materials with high functional properties. TO is a method which takes advantage of the high affinity of titanium and its alloys to oxygen. This method allows the surface layer to be modified by producing protective oxide films [10]. The mechanism and kinetics of the oxidation process are influenced by various factors including, but not limited to, chemical composition, temperature parameters and time of exposure [10,11]. The analysis of the course and mechanisms of the oxidation of titanium and its alloys is crucial to the process of obtaining high-quality oxide films. The thermal oxidation process can be divided into three main stages: oxygen adsorption, oxygen diffusion (oxygen atoms diffuse deep into the surface) and chemical reaction (oxygen reacts with titanium to form an oxide film). There is a mass gain on the surface of titanium materials during oxidation, the intensity of which depends on the temperature and time parameters. An appropriate selection of thermal oxidation process parameters results in a significant improvement in the performance characteristics of titanium materials, which is important in both engineering and biomedical applications [12,13,14].

Ti-6Al-4V alloy is among the most popular titanium alloys. One of the variants of this alloy is the Ti-6Al-4V ELI (Extra Low Interstitial) alloy. This alloy has a lower content of interstitial elements such as oxygen, carbon, nitrogen and hydrogen, which favorably affects the corrosion resistance. The reduction of the oxygen content (max. 0.13%) also results in improved ductility and crack resistance, with a slight reduction in strength compared to the Ti-6Al-4V alloy. The Ti-6Al-4V ELI alloy is therefore used in medical implants as, in addition to the required corrosion resistance, it is also characterized by greater biocompatibility [11,15].

In this study, the structural, micromechanical and tribological characteristics of the Ti-6Al-4V ELI alloy before and after TO were identified. The micromechanical tests were conducted to determine hardness, the mechanical work of indentation, the plastic deformation work, and the work of the elastic reverse deformation, as well as the elastic part of indentation work. Tribological tests were carried out in linear reciprocating motion in dry and lubricated contact (Ringer’s solution). Moreover, the impact of the oxidation temperature of the Ti-6Al-4 ELI alloy on surface roughness parameters was determined. The results of the experimental tests enabled the identification of key factors which influence the durability of oxide films deposited on the Ti-6Al-4V ELI alloy, which may directly enhance the applicability of the tested alloy.

## 2. Materials and Methods

In this study, the Ti-6Al-4V ELI alloy (α + β) was used. Examination of the chemical composition was conducted at Łukasiewicz Research Network—Upper Silesian Institute of Technology (Poland). The following research methods were used in the tests: ICP-OES (Inductively Coupled Plasma Optical Emission Spectrometry), HFIR and high-temperature extraction. The chemical composition of the Ti-6Al-4V ELI alloy is presented in Table 1.

The tests confirmed that the alloy’s chemical composition matched the specifications provided by the manufacturer. The primary alloying additions in the Ti-6Al-4V ELI alloy are aluminum and vanadium. These elements stabilize the α and β phases, respectively. Oxygen, in turn, increases the strength of titanium materials. However, compared to the Ti-6Al-4V alloy, the oxygen content in Ti-6Al-4V ELI was reduced to 0.12%, which has a beneficial effect on crack and plastic characteristics. The remaining elements are considered as impurities, and their content was also in compliance with the requirements (Table 1).

Ti-6Al-4V ELI alloy was subjected to thermal oxidation (TO) at 848 K, 898 K and 948 K over a period of 50 h. TO was carried out in a laboratory furnace in an air atmosphere. The parameters of the oxidation process were selected based on preliminary studies (oxidation kinetics). Following soaking at a predetermined oxidation temperature, the specimens were cooled in the furnace.

Observations of the surface microstructure of the Ti-6Al-4V ELI alloy after TO at 848 K, 898 K and 948 K were carried out using a JEOL JSM-6480 (JEOL, Tokyo, Japan), scanning electron microscope (magnification of 5000×).

Instrumental indentation testing of the Ti-6Al-4V ELI alloy was performed before and after TO using a Micro Combi Tester MCT^3^ (Anton Paar, Corcelles-Cormondrèche, Switzerland). Vickers indenter was used in the tests. Indentation curves were recorded at a maximum load of F_max_ = 200 mN. The loading and unloading time was determined in accordance with the recommendations of the ISO 14577-1 standard [16]. The hold time under maximum load was 10 s. For each specimen, 10 indentations were made. Based on the registered indentation curves, the values of Vickers hardness HV_IT_, the mechanical work W_total_, and its constituents: the plastic deformation work W_plast_, and the work of the elastic reverse deformation W_elast_, were determined. Furthermore, the elastic part of indentation work η_IT_ was calculated.

The mechanical work W_total_ performed during the loading and unloading of the indenter was partly dissipated on the plastic deformation work W_plast_, and partly stored as the work of the elastic reverse deformation W_elast_. Therefore, the mechanical work W_total_ was formulated as the sum of the plastic deformation work W_plast_, and the work of the elastic reverse deformation W_elast_ [17]:

(1) $$W_{total}=W_{plast}+W_{elast}=\int Fdx$$

**Figure 1:**
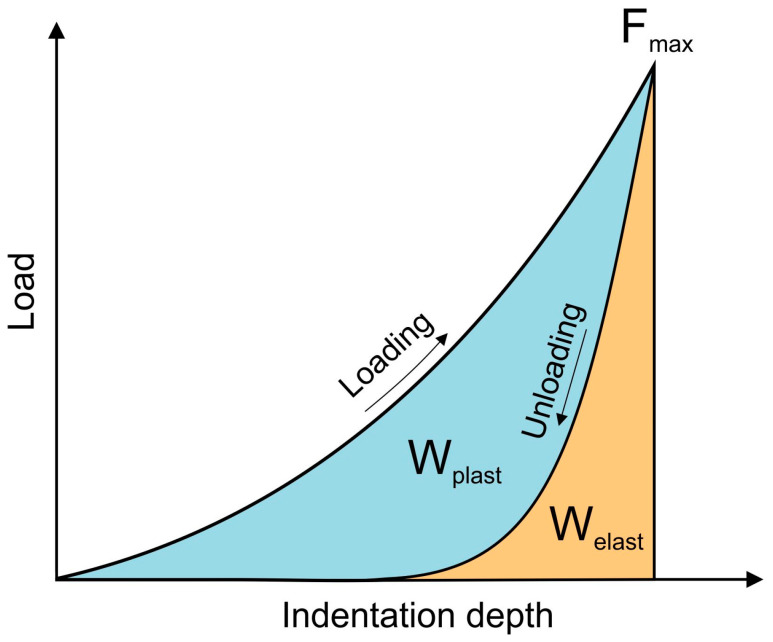
Plastic and elastic parts of the indentation work [16].

Both the plastic deformation work W_plast_, and the work of the elastic reverse deformation W_elast_ can be determined based on calculations of the surface area under the indentation curves (Figure 1).

According to standard ISO 14577-1, the elastic part of indentation work η_IT_ is calculated from the formula [16]:

(2) $$\eta_{IT}=\frac{W_{elast}}{W_{total}}\times 100$$

Examination of the tribological characteristics was carried out using a TRN Tribometer (Anton Paar, Corcelles-Cormondrèche, Switzerland). The tests were carried out in linear reciprocating motion in a dry contact and in Ringer’s solution. The solution was dosed in a fixed amount of 40 mL. The composition of the Ringer’s solution was as follows: sodium chloride (NaCl), potassium chloride (KCl), calcium chloride (CaCl_2_ × 6H_2_O) and water (H_2_O). The tests were performed at a load of 2 N load at 2 Hz frequency, over a friction distance of 50 m. The maximum linear velocity was 6.28 cm/s. The tests were performed at a temperature of 21 ± 1 °C and humidity of 40 ± 5%. ZrO_2_ balls of 6 mm in diameter were used as counter-specimens. The tests were performed in accordance with the recommendations of VAMAS and the ASTM G99 and G133 standards [18,19,20]. The static friction coefficient µ_s_ and dynamic friction coefficient µ_d_ were determined in the tribological tests conducted in the linear reciprocating motion.

The following formula was applied to determine the volume of the ball material (ZrO_2_) removed as a result of friction [21]:

(3) $$V=\pi h^{2}\left(r-\frac{h}{3}\right)$$

where:

- V—volume of the material removed during tribological tests [mm^3^],
- r—half-sphere radius [mm],
- h—the height of the wear volume [mm].

The h parameter was calculated using the following formula [21]:

(4) $$h=r-{\left(r^{2}-{\left(\frac{d}{2}\right)}^{2}\right)}^{\frac{1}{2}}$$

where:

- d—diameter of the wear trace of the ball [mm].

After tribological testing, the average surface area of the wear traces was assessed using a Surftest SJ-500 contact profilometer (Mitutoyo, Tokyo, Japan). Then, the specific wear rate (SWR) was determined using the formula:

(5) $$SWR=\frac{V}{L\cdot D}$$

where:

- SWR—specific wear rate [mm^3^/N·m],
- V—volume of material lost due to friction [mm^3^]; V = P·l, where P—average wear trace area, l—friction distance length (10 mm),
- L—load [N],
- D—friction distance [m].

Surface roughness tests of the Ti-6Al-4V ELI alloy before and after TO were carried out using the Surftest SJ-500 contact profilometer (Mitutoyo, Tokyo, Japan). Roughness was measured in accordance with the EN ISO 21920 standard using an elementary section and a measuring section selected appropriately for the surface roughness [22]. The tests determined the following parameters: Ra—arithmetic mean deviation of the assessed profile; Rq—root mean square deviation of the assessed profile; Rp—maximum profile peak height; Rv—maximum profile valley depth; Rz—maximum height of the profile; Rt—total height of the profile; Rsk—skewness of the assessed profile.

## 3. Results and Discussion

### 3.1. Surface Microstructure of the Ti-6Al-4V ELI Alloy after Thermal Oxidation

**Figure 2:**
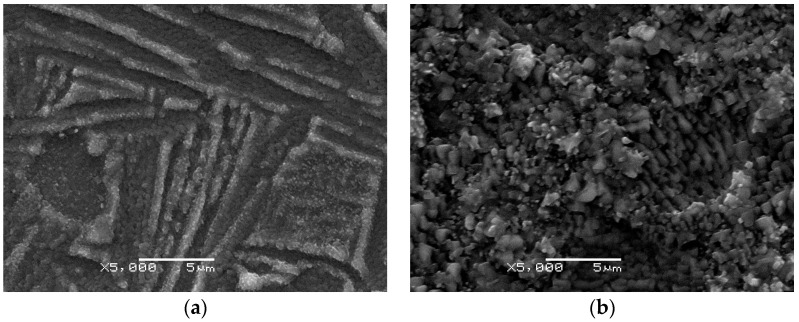

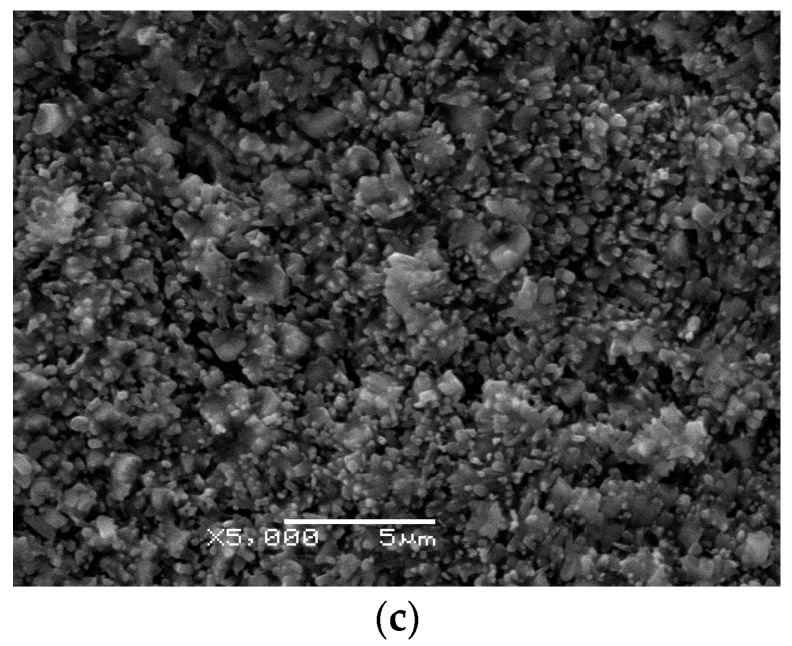
Surface microstructure of the Ti-6Al-4V ELI alloy after TO at: (**a**) 848 K, (**b**) 898 K, (**c**) 948 K.

SEM images of the surface microstructure of the Ti-6Al-4V ELI alloy after TO at 848 K, 898 K and 948 K are provided in Figure 2. The study revealed that oxide films fully covered the surface of the tested alloy and showed a progressive increase in oxide grain size at higher oxidation temperatures. It was also observed that increasing the oxidation temperature led to an improvement in the uniformity of the surface coverage of the Ti-6Al-4V ELI alloy with oxide layers. It was found that the oxidation temperature had a crucial effect on the morphological characteristics of the oxide films. The morphology of the oxide layers produced on the Ti-6Al-4V ELI alloy also depended on other (less significant) factors, such as: the chemical composition of the material (the presence of alloying additions, e.g., aluminum and vanadium, influenced the oxidation kinetics, which in turn shaped the morphology of the oxide films), the chemical composition of the furnace atmosphere, the surface preparation of the specimens and the cooling after oxidation (the specimens in this study were cooled in the furnace).

Changes in the oxide grain size and number as a function of temperature illustrate the mechanism of oxide layer formation. During oxidation at 848 K, the surface of the Ti-6Al-4V ELI alloy starts to absorb oxygen particles from the atmosphere, and fine oxide grains, characterized by a compact structure, are formed. A similar mechanism of oxide film formation on the twin Ti-6Al-4V alloy was observed by Wang et al. [23]. Due to the low intensity of the oxidation process, the oxide film that formed covered the surface of the Ti-6Al-4V ELI alloy in an irregular manner. Microscopic images revealed the presence of clusters of oxide particles, which were mainly concentrated in longitudinal areas of approximately 0.5–0.7 µm in width. Outside these areas, the presence of very fine oxide particles was observed (Figure 2a). TO carried out at 898 K resulted in higher uniformity in the surface coverage of the Ti-6Al-4V ELI alloy with oxide layers (Figure 2b). The most uniform coverage with oxide films was noted after oxidation at 948 K (Figure 2c). An increase in the uniformity of coverage with oxide particles with increasing oxidation temperature was also observed for other titanium alloys [12,24]. Simultaneously, it was observed that as the oxidation temperature rose, small oxide grains underwent a growth process, and large grains were thus formed. The large oxide grains were characterized by a high degree of agglomeration, which resulted in a high surface roughness of the films that formed (Section 3.5).

### 3.2. Micromechanical Tests

**Figure 3:**
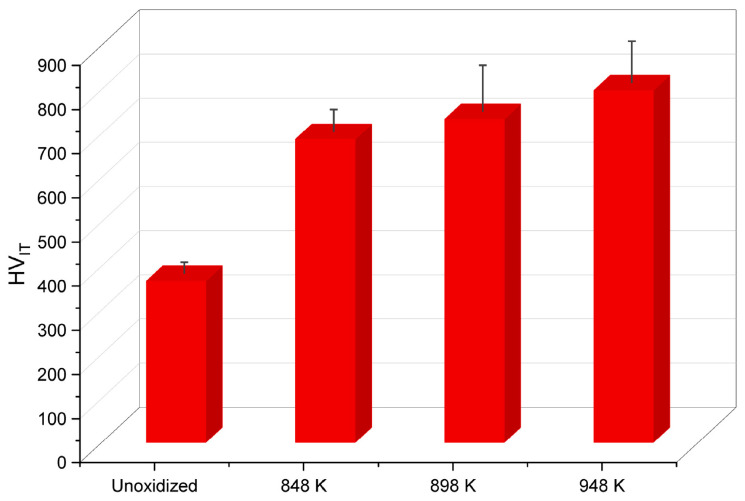
Hardness HV_IT_ before and after TO of the Ti-6Al-4V ELI alloy at 848 K, 898 K and 948 K.

In the next stage of the experimental studies, the micromechanical properties of the Ti-6Al-4V ELI alloy were determined before and after TO at 848 K, 898 K and 948 K. Figure 3 shows the HV_IT_ hardness measurements of the examined alloy, both in its as-received state and as a function of the TO temperature.

The study demonstrated that the Ti-6Al-4V ELI alloy in the as-received condition had the lowest hardness (366 HV_IT_). After TO, an increase in hardness HV_IT_ of the alloy was observed. As a result of oxidation of the Ti-6Al-4V ELI alloy at 848 K, its hardness increased to 687 HV_IT_ (an increase of approximately 88%). Raising the temperature to 898 K resulted in a further increase in the hardness of the material, reaching 733 HV_IT_ (an increase of approximately 100%). The greatest increase in hardness of the Ti-6Al-4V ELI alloy (798 HV_IT_) was observed after the oxidation process conducted at 948 K (an increase of approximately 118%). It was found that TO carried out at elevated temperatures led to an intensification of the diffusion processes, thereby resulting in the formation of hard oxide phases on the surface of the Ti-6Al-4V ELI [25,26].

**Figure 4:**
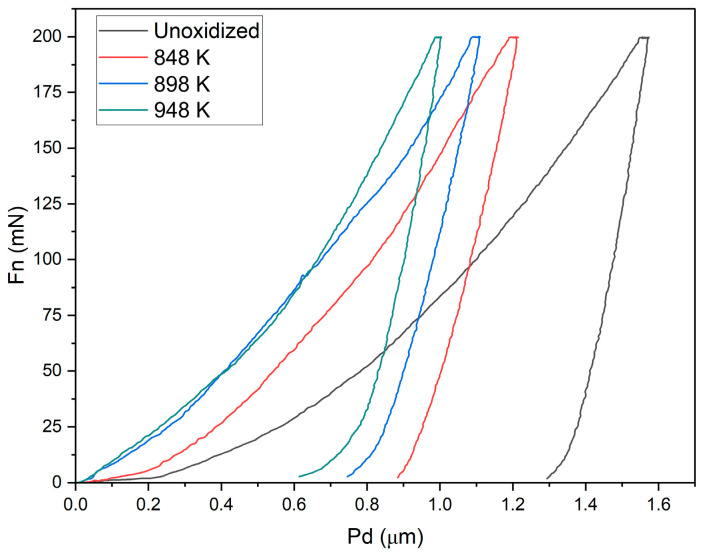
Examples of indentation curves presenting the indenter penetration depth (Pd) as a function of load (Fn) obtained after micromechanical tests of the Ti-6Al-4V ELI alloy in the as-received condition and after TO at 848 K, 898 K and 948 K.

Figure 4 illustrates examples of indentation curves before and after TO of the Ti-6Al-4V ELI alloy at 848 K, 898 K and 948 K.

The course of the indentation curves (P-h) was variable and dependent on the TO temperature of the Ti-6Al-4V ELI alloy. The greatest depth of indenter penetration (Pd) was found for the alloy in the unoxidized state. It was demonstrated that the penetration depth of the indenter (Pd) decreased with rising oxidation temperature. The test results obtained were consistent with the hardness measurements presented in Figure 3.

**Figure 5:**
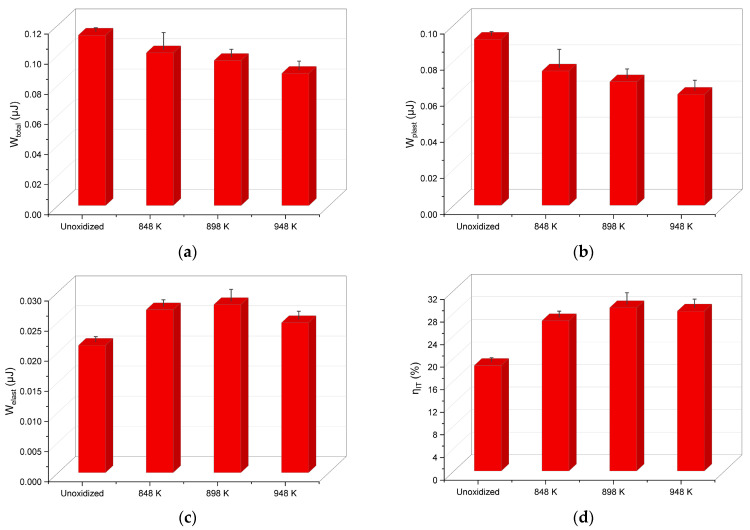
The mechanical work W_total_ (**a**), the plastic deformation work W_plast_ (**b**), the work of the elastic reverse deformation W_elast_ (**c**) and elastic part of indentation work η_IT_ (**d**) of the Ti-6Al-4V ELI alloy as-received, and after TO at 848 K, 898 K and 948 K.

Figure 5 presents changes in the work of indentation of the Ti-6Al-4V ELI alloy before and after TO (W_total_, W_plast_, W_elast_), and the elastic part of indentation work, η_IT_.

Based on the course of instrumental indentation curves, the mechanical work W_total_ and its constituents (the plastic deformation work W_plast_ and the work of the elastic reverse deformation W_elast_) were determined. The results of the analysis indicated that the unoxidized alloy had the lowest deformation resistance. It was shown that raising the TO temperature of the Ti-6Al-4V ELI alloy resulted in greater deformation resistance. This increase was observed, inter alia, through a decrease in the penetration depth of the indenter, which was directly reflected in a decrease in the value of the mechanical work, W_total_ (Figure 5a). It was found that W_total_ decreased with the rise of the oxidation temperature (a decrease of approximately 10–22% compared to the material as-received). A similar trend was observed for the plastic deformation work W_plast_ (max. decrease of approx. 33%)—Figure 5b. The study found that the TO of the Ti-6Al-4V ELI alloy performed at temperatures of 848 K and 898 K led to an increase in the work of the elastic reverse deformation W_elast_ (max. increase of approx. 32%). Only after oxidation at 948 K was a decrease in the value of the W_elast_ parameter observed, which could be related to the formation of an oxide film with high hardness and thus, a lower susceptibility to elastic deformation (Figure 5c). A similar dependency was reported for the elastic part of indentation work, η_IT_ (Figure 5d).

### 3.3. Tribological Tests

**Figure 6:**
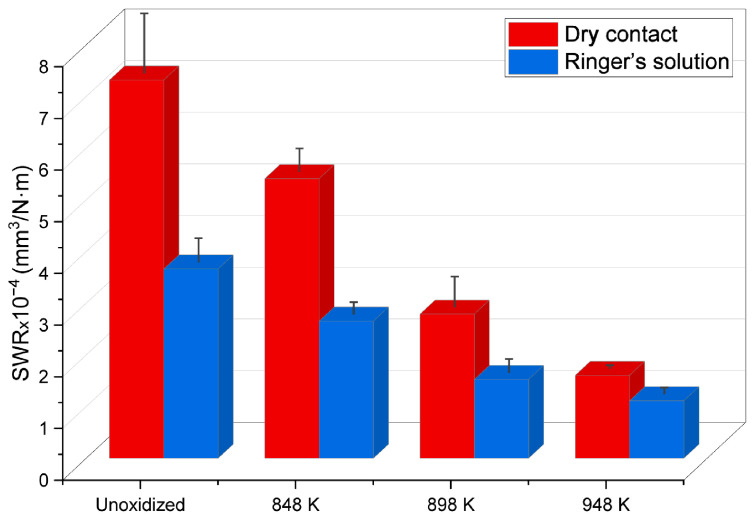
Specific wear rate (SWR) of the Ti-6Al-4V ELI alloy before and after TO at 848 K, 898 K and 948 K, after tribological tests carried out in dry and lubricated contact (Ringer’s solution).

Tribological tests of the Ti-6Al-4V ELI alloy as-received and after TO were carried out under dry friction conditions and with lubrication (Ringer’s solution). The tribological test results (specific wear rate) of the Ti-6Al-4V ELI alloy before and after oxidation at 848 K, 898 K and 948 K are provided in Figure 6.

The tribological tests conducted under dry friction conditions revealed that the Ti-6Al-4V ELI alloy in its as-received state exhibited the lowest resistance to sliding wear. After TO, a significant improvement of the tribological characteristics of the test material was observed. It was demonstrated that tribological resistance increased as the oxidation temperature rose (Figure 6). The oxide film obtained at 948 K had the highest sliding wear resistance (SWR reduction of approximately 78% compared to the unoxidized Ti-6Al-4V ELI alloy).

The improvement in the tribological properties of the Ti-6Al-4V ELI alloy after TO was related to the formation of hard oxide layers with specific tribological properties. The primary oxide in this type of film is TiO_2_ oxide characterized by high hardness and sliding wear resistance [10,14,23]. The presence of oxide films led to a limitation of plastic deformation and thus a reduction in the volume of material lost due to friction [27]. Furthermore, the oxide films that developed on the surface of the Ti-6Al-4V ELI alloy functioned as a protective barrier that reduced direct contact between the materials interacting tribologically [28]. Another factor in the wear reduction was a lower tendency to adhesion after oxidation, which lowered the risk of cold welding during tribological contact (the oxide layer reduced the adhesion forces between the friction surfaces) [23,26].

More favorable tribological test results were obtained under lubrication conditions (Ringer’s solution) as compared to tests in dry contact. For the Ti-6Al-4V ELI alloy in its unoxidized state, the determined reduction in SWR was approximately 50% in relation to tests under dry friction conditions. After TO, the sliding wear resistance of the Ti-6Al-4V ELI alloy in the lubricated contact also increased with the rise in oxidation temperature. However, when compared to dry contact tests, a more significant wear reduction was observed for each temperature variant analyzed. After TO of the Ti-6Al-4V ELI alloy at 848 K and 898 K, SWR values were approximately 50% lower in comparison to tests without lubrication. Tribological tests carried out on specimens oxidized at 948 K showed a slightly lower reduction in wear compared to dry contact tests (by about 30%). Of all the specimens tested, the most favorable results of the tribological test were obtained at this temperature variant (948 K) (Figure 6). The improvement in tribological characteristics after tests in Ringer’s solution was related to the presence of an additional medium which contributed to the formation of a lubricating film between the interacting surfaces [29]. Thus, a thin layer was formed which separated the interacting surfaces, minimizing direct contact between the examined alloy and the ZrO_2_ balls.

**Figure 7:**
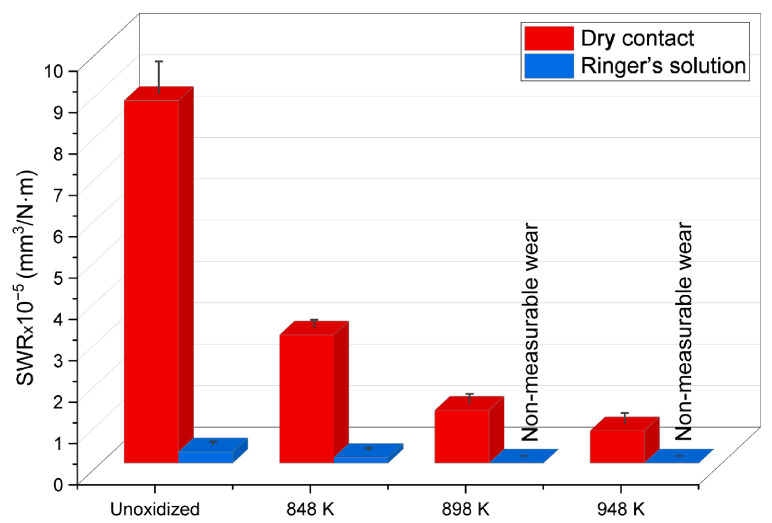
Specific wear rate (SWR) of ZrO_2_ balls after tribological tests in dry and lubricated contact (Ringer’s solution) with the Ti-6Al-4V ELI alloy as-received, and after TO at 848 K, 898 K and 948 K.

Figure 7 presents the specific wear rate (SWR) of ZrO_2_ balls used as counter-specimens in tribological tests.

The tests showed that ZrO_2_ balls were worn most severely in the non-lubricated contact when tested in a friction couple with the non-oxidized Ti-6Al-4V ELI alloy. The sliding wear resistance of the ZrO_2_ balls improved as the oxidation temperature of the tested alloy increased. The greatest reduction in the wear of the ZrO_2_ balls during dry contact tests was found after interaction with the titanium alloy oxidized at 948 K (SWR reduction of approximately 91%), whereas, after tribological tests in Ringer’s solution, the reduction in wear of the ZrO_2_ balls was significantly greater. In a friction pair with the non-oxidized Ti-6Al-4V ELI alloy and after oxidation at 848 K, the SWR value decreased by approximately 98%. For the remaining temperature variants (898 K, 948 K), the wear of the balls was unmeasurable under the adopted test conditions (Figure 7).

The formation of protective layers on the Ti-6Al-4V ELI alloy was crucial for reducing the ZrO_2_ ball wear. These layers significantly improved the tribological properties of the entire friction system, primarily by reducing adhesion. Additionally, oxides such as TiO_2_ can function as a solid lubricant, which also led to wear reduction. The Ringer’s solution, in turn, served as a lubricant and protective agent, which improved the friction conditions of the ZrO_2_ balls, minimizing their wear and tear in comparison to the dry tests [30].

### 3.4. Friction Coefficient

**Figure 8:**
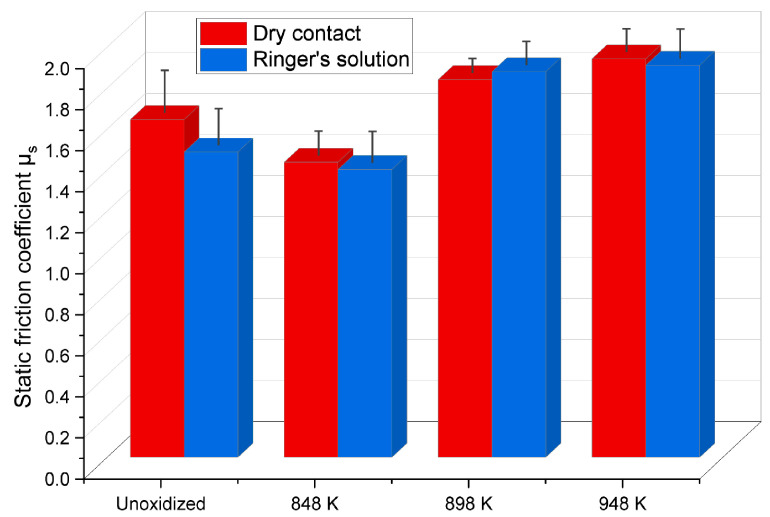
Static friction coefficient µ_s_ of a Ti-6Al-4V ELI-ZrO_2_ friction couple during tribological tests in dry and lubricated contact (Ringer’s solution).

**Figure 9:**
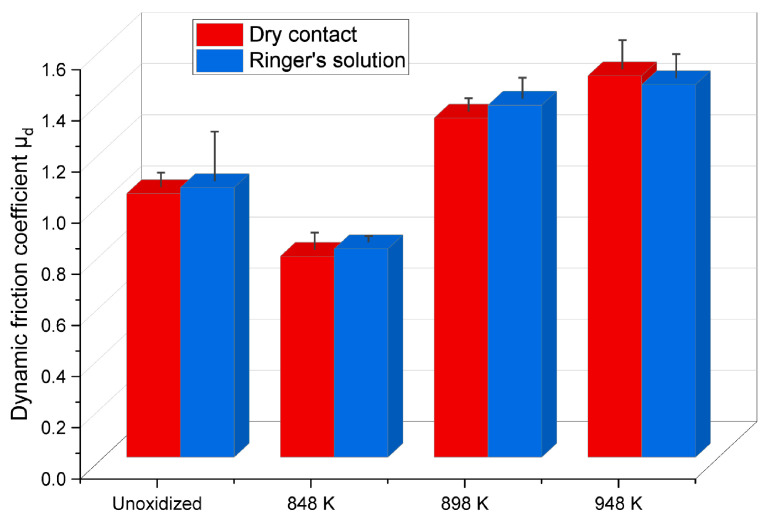
Dynamic friction coefficient µ_d_ of the Ti-6Al-4V ELI-ZrO_2_ friction couple during tribological tests in dry and lubricated contact (Ringer’s solution).

In tribological tests in linear reciprocating motion, the values of the static friction coefficient µ_s_ and dynamic friction coefficient µ_d_ of the Ti-6Al-4V ELI-ZrO_2_ friction couple were determined in both dry and lubricated contact (Ringer’s solution). The results are shown in Figure 8 and Figure 9.

The tests demonstrated that the static and dynamic friction coefficients (µ_s_, µ_d_) reached high values during tribological tests in both dry and lubricated contact (Ringer’s solution), but this did not result in an increase in wear severity. For the non-lubricated contact, it was observed that the static friction coefficient, µ_s_, for the Ti-6Al-4V ELI alloy in its unoxidized state, when tested with ZrO_2_ balls, was high, i.e., approximately 1.6. The thermal oxidation process of the Ti-6Al-4V ELI alloy at 848 K reduced the friction coefficient value of the friction couple analyzed to 1.4, which was associated with the formation of a thin oxide film (acting as a solid lubricant) with a low surface roughness. At higher oxidation temperatures (898 K, 948 K), a repeated increase of the static friction coefficient µ_s_ (to approx. 1.8) was observed. Following tests in a Ringer’s solution, similar values of the static friction coefficient µ_s_ were obtained. For the Ti-6Al-4V ELI (non-oxidized)-ZrO_2_ friction couple, the static friction coefficient µ_s_ value was slightly lower (1.5) compared to dry contact tests. A reduction in the friction coefficient value (1.4) was also observed when testing the Ti-6Al-4V ELI alloy after oxidation at 848 K. However, after oxidation at 898 K and 948 K, the static friction coefficient µ_s_ increased to a value of about 1.9, indicating similar mechanisms of tribological interaction to those found in dry friction. At the same time, no reduction in the static friction coefficient µ_s_ was observed when tested in the Ringer’s solution.

The dynamic friction coefficient µ_d_ reached lower values than the static friction coefficient µ_s_, which is typical once the static friction resistance has been overcome. The dynamic friction coefficient µ_d_ of the Ti-6Al-4V ELI (non-oxidized)-ZrO_2_ friction couple during dry contact tests was approximately 1.0. After oxidation at 848 K, the dynamic friction coefficient µ_d_ was observed to decrease to approximately 0.8. The obtained results indicate a synergistic effect of the oxide layer with low surface roughness on the reduction of the dynamic friction coefficient µ_d_. After oxidation at higher temperatures (898 K, 948 K), the coefficient µ_d_ increased to values of 1.3 and 1.5 respectively, which was related, in a similar manner to the case of static friction, to the increase in surface roughness of the Ti-6Al-4V ELI alloy. The tests found no effect of the Ringer’s solution on the reduction of the dynamic friction coefficient µ_d_.

An analysis of the roughness parameters (Section 3.5) revealed significant changes in the surface topography after oxidation of the Ti-6Al-4V ELI alloy at 898 K and 948 K, as a result of the growth and agglomeration of oxide grains. These changes led to the formation of irregularities on the surface, which consequently increased the value of the static and dynamic friction coefficients, µ_s_ and µ_d_, respectively [23,31]. In addition, the oxide layers obtained after oxidation of the alloy at 898 K and 948 K showed a tendency towards greater adhesion in contact with the ZrO_2_ balls, which also resulted in an increased friction force.

The study found that TO at higher temperatures (898 K, 948 K) increased the static (µ_s_) and dynamic (µ_d_) friction coefficients, while reducing wear. Thus, there was no significant correlation identified between the results of the wear tests (Figure 6 and Figure 7) and the friction coefficient (Figure 8 and Figure 9). An analysis of literature data on the effects of the TO of titanium and its alloys on the wear and friction values revealed significant discrepancies in this respect, probably due to the complex nature of friction, the various materials used in the friction couples and changing test conditions [32,33,34]. In the research presented in this paper, it was demonstrated that the wear reduction of the Ti-6Al-4V ELI alloy after TO was more influenced by factors other than the friction coefficient, such as the morphology, hardness and surface topography of the oxide films. At the same time, tribological tests in a lubricated contact revealed that the lubricating medium (Ringer’s solution) also significantly enhanced the tribological characteristics.

### 3.5. Surface Roughness

**Figure 10:**
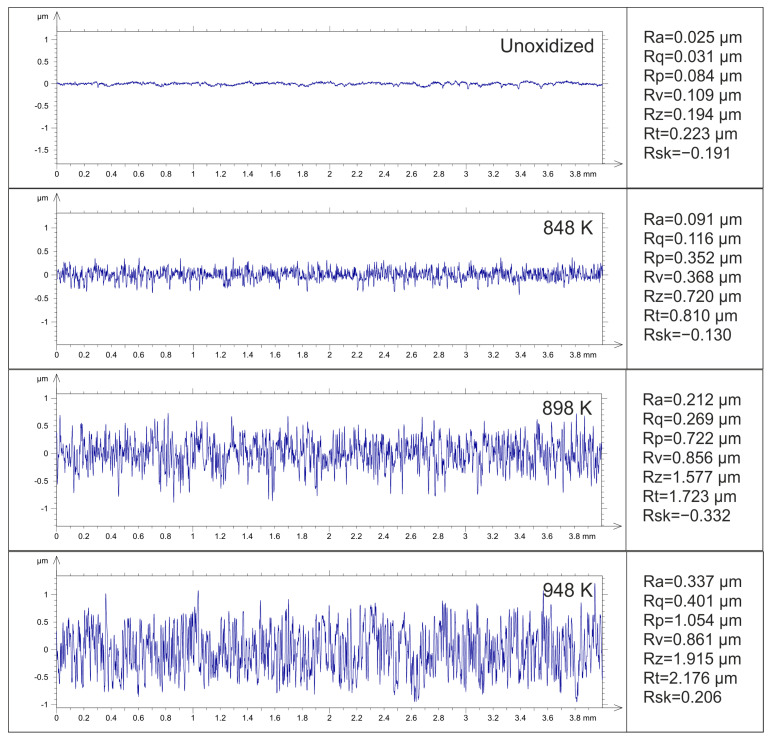
Examples of surface outline profiles and selected roughness parameters before and after TO of Ti-6Al-4V ELI alloy at 848 K, 898 K and 948 K.

The thermal oxidation process, which involves the controlled exposure of the Ti-6Al-4V ELI alloy to high temperatures in an oxidizing atmosphere, results in the development of oxide layers on its surface which considerably alter the surface roughness and topography. Figure 10 presents examples of surface outline profiles and selected roughness parameters before and after TO of the Ti-6Al-4V ELI alloy.

The surface of the Ti-6Al-4V ELI alloy before oxidation was characterized by low surface roughness (Ra = 0.025 µm). After TO of the Ti-6Al-4V ELI alloy at 848 K, the surface roughness parameters (Ra = 0.091 µm) were only slightly higher than the values obtained for the non-oxidized material. At elevated oxidation temperatures, the formation of oxide layers on the Ti-6Al-4V ELI alloy led to an increase in surface roughness (898 K—Ra = 0.21 µm; 948 K—Ra = 0.34 µm). The increase in surface roughness parameter values was associated with an increase in the thickness of the oxide films and the formation of oxide grains of a larger size [23].

**Figure 11:**
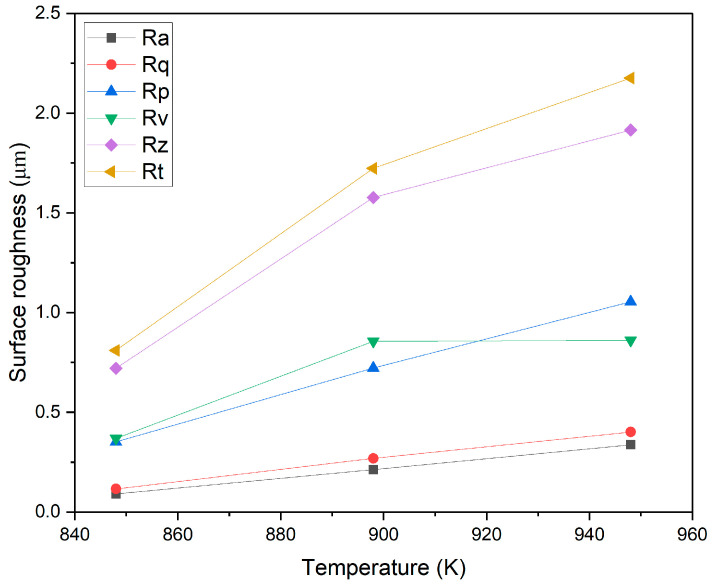
Effect of TO temperature on the roughness of oxide films forming on Ti-6Al-4V ELI alloy.

Figure 11 illustrates the influence of oxidation temperature on the surface roughness characteristics of the Ti-6Al-4V ELI alloy. It was found that an increase in oxidation temperature from 848 K to 948 K led to a linear increase in parameters such as Ra, Rq and Rp. The value of parameters such as Rz and Rt also increased with increasing oxidation temperature. The oxidation temperature was found to have a minor effect on the increase in the value of the Rv parameter only.

## 4. Conclusions

The thermal oxidation process effectively enhances the micromechanical and tribological properties of the Ti-6Al-4V ELI alloy. The study shows that modifying the oxidation temperature can shape the microstructural features and functional properties of the oxide films formed on the Ti-6Al-4V ELI alloy. As the oxidation temperature increased, the uniformity of coverage improved, and the grain size of the oxides being formed increased. Micromechanical tests showed that the Ti-6Al-4V ELI alloy had increased hardness after thermal oxidation. It was therefore concluded that increasing the thermal oxidation temperature led to an increase in deformation resistance. After thermal oxidation of the Ti-6Al-4V ELI alloy, a decrease was found in the mechanical work W_total_, by approximately 10–22% compared to the as-received material. A similar tendency was observed for the plastic deformation work W_plast_ (max. decrease by approx. 33%). In tribological tests, it was found that the wear resistance of the Ti-6Al-4V ELI alloy increased with the rise of the oxidation temperature both for dry contact friction and in the Ringer’s solution. It was demonstrated that the Ringer’s solution had a beneficial effect on reducing the specific wear rate (wear reduction of approximately 30–50% compared to dry contact tests). Under both dry friction and lubricated contact conditions, the lowest wear rates were achieved after oxidation of the Ti-6Al-4V ELI alloy at 948 K. In addition, it was found that the reduced specific wear rate of Ti-6Al-4V ELI after thermal oxidation was the result of a synergistic effect of several factors. The resulting oxide film increased hardness, improved chemical stability and reduced the propensity for adhesion. Tribological tests also revealed the beneficial effect of the thermal oxidation process of the Ti-6Al-4V ELI alloy on the reduction of wear of the ZrO_2_ balls used as counter-specimens. Another conclusion is that the oxidation temperature enabled control of the geometrical surface structure parameters (roughness) of the Ti-6Al-4V ELI alloy. It was demonstrated that the surface roughness increased with the increase in temperature. This is particularly important for biomedical applications, where surface roughness has a significant impact on, inter alia, tissue integration and the sustainability of implants, which is crucial for their clinical efficacy. The findings clearly demonstrate the potential of thermal oxidation as an effective method for improving the micromechanical and tribological characteristics of the Ti-6Al-4V ELI alloy.

## Figures and Tables

**Table 1:** Chemical composition of Ti-6Al-4V ELI alloy.

	Components Content, wt. %.						
	Al	Fe	N	O	C	V	Ti
Limit	5.5–6.5	max 0.25	max 0.05	max 0.13	max 0.13	3.5–4.5	Remainder
Result	6.09	0.22	0.02	0.12	0.021	4.11	Remainder

## Data Availability

The original contributions presented in the study are included in the article, further inquiries can be directed to the corresponding author.
